# Single-cell pigment analysis of phototrophic and phyllosphere bacteria using simultaneous detection of Raman and autofluorescence spectra

**DOI:** 10.1128/aem.00129-25

**Published:** 2025-04-10

**Authors:** Nanako Kanno, Shinsuke Shigeto

**Affiliations:** 1 Department of Chemistry, School of Science, Kwansei Gakuin University 204978, Sanda, Hyogo, Japan; University of Tennessee at Knoxville, Knoxville, Tennessee, USA

**Keywords:** resonance Raman spectroscopy, carotenoids, bacteriochlorophyll *b*, purple phototrophic bacteria, aerobic anoxygenic phototrophic bacteria, *Methylobacterium*, autofluorescence

## Abstract

Microbes produce various types of pigments that are essential for their biological activities. Microbial pigments are important for humans because they are used in the food industry and medicine. The visualization and evaluation of the pigment diversity of microbial cells living in natural environments will contribute not only to the understanding of their ecophysiology but also to the screening of useful microbes. Here, we demonstrate the simultaneous, nondestructive detection of the resonance Raman and autofluorescence spectra of pigments in model purple phototrophic bacteria at the single-cell level. The single-cell Raman spectra measured using confocal laser Raman microspectroscopy with 632.8 nm excitation covered the wavenumber range of 660–3,022 cm^−1^ (corresponding to 661–783 nm), in which the autofluorescence spectra from the pigments can be detected simultaneously as a baseline. The peak position of the resonance Raman spectra of the carotenoids in the cells provided information on the length of the polyene chain and structural characteristics, such as conjugated keto groups and terminal rings. By contrast, the extracted autofluorescence spectra of purple phototrophic bacteria differed in pattern depending on bacteriochlorophyll type (*a* or *b*), suggesting that their autofluorescence originates from bacteriochlorophyll-related molecules. In addition, we revealed the pigment diversity in microbial cells on the leaf surface and isolated pigmented bacteria that could contribute to the pigment diversity of the environmental sample. Our study shows that Raman and fluorescence microspectroscopy is a useful tool for finding novel pigmented microbes and uncovering yet unknown relationships between microbes and light.

**IMPORTANCE:**

To understand the activities of microbes in natural environments, it is important to know the types of biomolecules they express *in situ*. In this study, we report a method using resonance Raman and autofluorescence signatures to detect and distinguish the types of carotenoid and bacteriochlorophyll pigments in intact, living cells. We have shown that this method can be used to estimate the expression status and pigment types in purple phototrophic bacteria and carotenoid-producing bacteria as well as the diversity of the pigments expressed by microbes on the leaf surface. Our method requires little pretreatment and can analyze pigments without destroying cells, making it a useful tool for visualizing phototrophic activity and searching for unidentified microbes.

## INTRODUCTION

Microbes can produce various types of pigments, such as carotenoids, porphyrins, and bilins, that correlate with light energy harvesting, photosensing, and stress resistance. In the fields of food and human health, these pigments are used as coloring agents and functional supplements (1, 2). The visualization and evaluation of pigment diversity in environmental microbes should contribute not only to the understanding of microbial ecophysiology but also to the screening of useful microbes.

Anoxygenic phototrophic bacteria harboring bacteriochlorophylls (BChls) and carotenoids are well adapted to the sunlit environment of the Earth. They are found in freshwater, marine, and extreme environments, such as hot springs (3), saline environments (4), and polar regions (5, 6). Although light utilization by phototrophic bacteria has been well studied in laboratory-based studies, their distribution and light utilization in natural environments are still unknown. One of the reasons for this is the difficulty in the discrimination of phototrophic bacteria by gene-based bacterial community analysis. The expression of photosynthetic pigments is regulated by various environmental conditions, such as oxygen, light, and nutrient availability (7, 8), and most phototrophic bacteria can grow both phototrophically and chemotrophically. The phylum *Pseudomonadota* contains a large number of phototrophic bacteria known as purple phototrophic bacteria. They are widely distributed throughout the phylum, but due to repeated deletions and horizontal gene transfer of photosynthetic genes during the evolution of bacteria belonging to the phylum *Pseudomonadota*, phylogenetic trees based on 16S rRNA genes and photosynthetic genes are not always consistent (9). Furthermore, several new phyla of bacteria, including phototrophic bacteria, have been reported in the past decade (10, 11). Therefore, genetic analysis alone does not provide sufficient information to assess the distribution and ecophysiology of phototrophic bacteria in the environment and must be used in combination with the evaluation of pigment expression.

Fluorescence microscopy and Raman microspectroscopy have been used to study photosynthetic pigments. Cells containing BChl *a* or carotenoids are detected by near-infrared fluorescence microscopy (12–14) or Raman microspectroscopy, respectively (15–17). Because the absorption and fluorescence peaks of BChl *a* are red-shifted compared with chlorophyll *a* in cyanobacteria and higher plants, these fluorescence characteristics are used to detect phototrophic bacteria. Carotenoids are one of the biomolecules that can be easily detected from cells using Raman microspectroscopy, which is a nondestructive and label-free method for obtaining biomolecular information as a Raman spectrum from single microbial cells. When a laser beam is incident on a sample, most of the scattered light has the same wavelength as incident light, but the wavelength of a small fraction of the scattered light is shifted by the vibrational energy of the molecules, which is called Raman scattered light. The resonance Raman effect occurs when the wavelength of the incident light is close to the absorption band of a pigment, and the intensity of the Raman peaks of the pigment is enhanced significantly (18). The resonance Raman spectra of the carotenoids had strong Raman peaks at ~1,000, 1,150, and 1,500 cm^−1^, which are assigned to the C–CH_3_, C–C, and C = C vibrational modes of the conjugated chains in carotenoids, respectively. The peak position at ~1,500 cm^−1^ correlates with the number of conjugated C = C bonds, *N* (18, 19), but varies depending on the structure of the carotenoid and the surrounding environment (20–23). Therefore, it is crucial to directly measure the Raman spectra of carotenoids inside cells in a nondestructive manner. Because both Raman scattering and fluorescence occur at wavelengths longer than the wavelength of the incident light, the Raman spectrum of a fluorescent sample with sharp Raman peaks is almost always accompanied by a broad fluorescence spectrum unless it is excited at a longer wavelength (i.e., near-infrared). Although the two spectroscopic methods have advantages, the complementary information they provide is rarely utilized together—fluorescence is usually considered a vexing background that severely interferes with Raman signatures.

In this study, we demonstrate the simultaneous detection of the resonance Raman spectra of carotenoids and the autofluorescence spectra characteristic of model phototrophic bacteria at the single-cell level. The pigment spectra of the phototrophic bacterial cells—reflecting multiple types of intracellular pigments—were separated into autofluorescence and Raman component spectra. This methodology differs from conventional fluorescence microscopy in that it extracts the fluorescence spectrum, which can be used to compare spectral shapes. In addition to phototrophic bacteria, nonphototrophic and carotenoid-producing bacteria were examined, and the resonance Raman spectra of carotenoids were compared across all bacterial species studied. The comparison showed that the spectral shape reflects differences not only in carotenoid type but also in BChl type. This result suggests that our assay can be used to detect a wide range of phototrophic bacteria and to distinguish between the pigment compositions of bacterial cells, which vary depending on bacterial species and physiological states.

We evaluated the microbes that colonize the surface of plant leaves. These microbes are closely associated with the health and growth of the host plant (24, 25). The leaf surface environment, where sunlight falls, serves as a site of photosynthesis for microbes (12). However, it is a challenging environment for microbes, where they are exposed to UV radiation, temperature fluctuation, drought, and nutrient limitation, but a certain number of microbes are usually found to colonize. Several studies have reported the diversity of phototrophic bacteria based on gene analysis and detection of BChl *a* autofluorescence (12, 14, 26). However, it is unclear how many bacterial lineages with diverse photosynthetic genes suggested by the gene-level analysis exhibit phototrophic activity at the plant surface. Although a method has been used to pick up and culture cells that emit BChl autofluorescence (14), it is difficult to identify bacterial species by autofluorescence alone, resulting in a biased selection of microbial species that are relatively abundant in the phyllosphere. In this study, we addressed this issue by detecting the autofluorescence of microbial cells in the phyllosphere specific to phototrophic bacteria, in addition to the carotenoid pigments exhibited by fluorescent cells.

## MATERIALS AND METHODS

### Bacterial strains and culture conditions

In this study, we used seven strains of purple phototrophic bacteria (anaerobic and aerobic anoxygenic phototrophic bacteria), four strains of nonphototrophic and carotenoid-producing bacteria, and one strain that produced neither photosynthetic pigments nor carotenoids (Table S1). *Blastochloris viridis* NBRC 102659^T^, *Deinococcus radiodurans* NBRC 15346^T^, *Methylobacterium komagatae* NBRC 103627^T^, *Micrococcus luteus* NBRC 3333^T^, *Rhodobacter sphaeroides* NBRC 12203^T^, *Rhodospirillum rubrum* NBRC 3986, *Roseobacter litoralis* NBRC 15278^T^, *Rubrivivax gelatinosus* NBRC 100245, and *Sphingomonas astaxanthinifaciens* NBRC 102146^T^ were obtained from the National Institute of Technology and Evaluation (NITE) Biological Resource Center of Japan (NBRC). *Bacillus subtilis* JCM 1465^T^ and *Rhodococcus erythropolis* JCM 3201^T^ were obtained from the Japan Collection of Microorganisms (JCM). Cultured *Allochromatium vinosum* strain D was kindly provided by Dr. T. Shimizu.

The culture conditions for the phototrophic bacteria were as follows: *B. viridis, R. gelatinosus, R. rubrum*, and *R. sphaeroides* were anaerobically cultured in peptone-yeast-succinate (PYS) medium (pH 7.0) containing (per liter) 5 g disodium succinate hexahydrate (FUJIFILM Wako Pure Chemical Corp.), 5 g Bacto Peptone, 1 g Bacto Yeast Extract (Becton, Dickinson and Company, Franklin Lakes, NJ, USA), and 10 mL of a basal salt solution (27) at 30°C under a light-emitting diode (LED) light source emitting 850 nm radiation (ISL-150X150II85; CCS Inc., Kyoto, Japan). *M. komagatae* was aerobically cultured in 5 mL of R2A broth (Daigo; Nihon Pharmaceutical, Tokyo, Japan) at 30°C with shaking at 200 rpm, without illumination. Cells were collected from the early stationary to stationary phase. *R. litoralis* was aerobically cultured on PYS plates (1.5% agar) at 30°C without LED illumination, and the cells were harvested after 7 days of culture. Aerobically cultured (at mid-exponential phase) *R. sphaeroides* were obtained by culturing the cells in 30 mL volumetric test tubes containing 5 mL of PYS medium at 30°C and 200 rpm shaking conditions without illumination. Cells of *A. vinosum*, cultured anaerobically in minimal inorganic culture medium (28) with 850 nm LED illumination at 30°C, were incubated in our laboratory for 1 day under the same culture conditions after 1 day of transport between laboratories and then used for Raman measurements.

All nonphototrophic bacteria were aerobically cultured in liquid media (5 mL medium in 30 mL volumetric tubes) at 200 rpm shaking conditions without illumination, and the cells were collected from the early stationary to stationary phase. *D. radiodurans, M. luteus*, and *S. astaxanthinifaciens* were cultured in NBRC No. 702 medium at 30°C, 30°C, and 40°C, respectively. *R. erythropolis* was cultured in JCM No. 43 medium at 28°C. *B. subtilis* was cultured in BD Bacto Tryptic Soy Broth (Becton, Dickinson and Company) at 30°C.

The bacterial growth in the liquid culture was monitored by measuring optical density at 660 nm using a photometer (Miniphoto 518R; TAITEC Co., Saitama, Japan).

### Sampling and isolation procedure

Plant leaf samples were collected from white clover (*Trifolium repens*) growing outdoors in Sanda, Hyogo, Japan, in August 2022. Ten trifoliate leaves in 30 mL of phosphate-buffered saline (PBS, pH 7.4) were sonicated for 3 min in an ultrasonic bath (US-103; SND Co., Nagano, Japan). The supernatant was filtered through a 5 µm pore size cellulose acetate syringe filter (Sartorius AG, Göttingen, Germany). Spectral measurements were performed on the same day as the sampling.

For isolation, a portion of the filtrate was inoculated onto PYS plates, and pink, orange, and yellow colonies were transferred and streaked onto new plates several times to obtain pure cultures. Six microbial strains were isolated and named STP2, STP3, STO1, STY1, STY2, and STY3 (Table 1). Cells from all strains, excluding STP2 and STP3, were collected from PYS agar to obtain single-cell spectra. To measure the single-cell spectra of STP2 and STP3, the cells were cultured on 1.5% agar plates of R2A medium (Nihon Pharmaceutical, Tokyo, Japan) under a light/dark cycle (12/12 h) using a daylight fluorescent lamp (60 W) for 5 days.

**TABLE 1:** Resonance Raman peak positions, colony color, and identification based on the 16S rRNA gene sequence in the strains isolated from clover leaves

Isolate name	Colony color	Raman peak position (cm^−1^)^*a*^		Closest relative (accession no.)	16S rRNA gene identity (%)
		ν_1_	ν_2_		
STP2	Pink	1,507	1,152	*M. goesingense* (NR_115219.1)	99.2
STP3	Pink	1,506	1,151	*M. komagatae* (NR_041441.1)	99.4
STO1	Orange	1,519	1,156	*Sphingomonas palmae* (KX893410.1)	99.9
STY1	Yellow	1,523	1,157	*Sphingomonas yabuuchiae* (NR_028634.1)	99
STY2	Yellow	1,522	1,156	*Curtobacterium luteum* (NR_026157.1)	99.5
STY3	Yellow	1,531	1,133	*Chryseobacterium ginsenosidimutans* (NR_108691.1)	98.5

^*a*^ Peak positions were calculated as an average of five cells for each strain.

### Single-cell Raman and autofluorescence spectroscopy

Single-cell Raman and autofluorescence spectra were measured using a laboratory-built confocal Raman microspectrometer equipped with an inverted microscope [TE2000-U (customized); Nikon Co., Tokyo, Japan], as described (29–32). The 632.8 nm output of a He–Ne laser (HNL210L; Thorlabs Inc., Tampa, FL, USA) was used as excitation light. Although we attempted 532 nm excitation, which is usually used to detect the resonance Raman spectra of carotenoids, we were unable to obtain reproducible pigment spectra due to rapid photobleaching (33, 34). The excitation wavelength of 632.8 nm did not overlap with the defined absorption peaks of BChls *a* and *b* (Q_x_ absorption peak: 590–605 nm; Q_y_ peak: >800 nm) and carotenoids (400–600 nm) found in phototrophic bacterial cells. The beam was focused onto the sample with an oil-immersion phase contrast objective [CFI Plan FluorDLL (100×, NA 1.3); Nikon], and backward Raman scattered light was collected with the same objective. An ordinary, non-phase contrast objective, as used in our studies (35), would have been a better choice of objective in terms of signal acquisition, but in this study, the phase contrast objective was employed to clearly observe the shape of prokaryotic cells, including those in the environmental sample. After passing it through a 100 µm pinhole for confocal detection, the Raman scattered light was analyzed with an imaging spectrometer (MS3504i; SOL Instruments, Minsk, Belarus) with 600 grooves mm^−1^ grating and detected using an electron-multiplying charge-coupled device detector with 1,600 pixels (DU970P-BVF; Andor Technology Ltd., Belfast, United Kingdom). All Raman spectroscopy measurements were performed in the wavenumber range of 660–3,022 cm^−1^ (corresponding to 661–783 nm) with a spectral resolution of ~5 cm^−1^. The laser power at the sample point was adjusted to 1 mW.

The workflow of our single-cell Raman and autofluorescence measurements and data preprocessing is shown in Fig. S1. Cells of pure cultures were washed three times with PBS by centrifugation (8,000–10,000 × *g*, 30–60 s, room temperature) and resuspended in PBS. The cell suspension was transferred to a glass-bottomed dish (Matsunami Glass Ind. Ltd., Osaka, Japan) or a quartz glass-bottomed dish (Fine Plus International Ltd., Kyoto, Japan). Optical tweezers utilize a laser beam focused by a high-numerical aperture (NA) microscope objective lens to capture microscopic objects at the focal point (36, 37) and can capture a cell in PBS. This technique is frequently employed in Raman measurements of microbial cells (38–41). The same laser can be employed for both optical tweezers and Raman measurements. Optical tweezers allow individual microbial cells to be captured in PBS at a depth away from the surface of the glass substrate, enabling the acquisition of the Raman spectra of the cell without interference from the glass background. For each strain of phototrophic bacteria and nonphototrophic and carotenoid-producing bacteria, the spectra of 25 optically trapped cells were measured. A series of spectra was measured every 30 s of exposure time up to 90 s, resulting in three consecutive spectra.

For plant leaf samples, a portion of the filtrate (1 mL volume in three 1.5 mL microtubes) was centrifuged (10,000 × *g*, 3 min, 4°C), concentrated 15-fold, and washed twice (10,000 × *g*, 1 min, 4°C) with 200 µL of PBS. The cell suspension was transferred to a quartz glass bottom dish, and spectra (every 30 s of exposure time up to 90 s) were measured from 100 cell-like particles (<5 µm) on the quartz coverslip. Prior to the measurement, the shape of the particles at the bottom of the quartz dish was verified using phase contrast imaging.

For comparison with phototrophic bacteria and carotenoid-producing bacteria, single-cell spectra of *B. subtilis* (nonphototrophic and noncarotenoid-producing bacteria) were measured from 25 cells that were optically trapped or on the quartz coverslip.

### Raman-autofluorescence spectral analysis

The spectral sensitivity of all recorded spectra was calibrated using the spectrum of a standard halogen light source (HL-3 plus; Ocean Insight, Orlando, FL, USA). The PBS spectrum (average of 10 spectra) was subtracted from each cell spectrum acquired with laser trapping. For the spectrum obtained from the cells on the quartz glass, the averaged spectrum measured outside the cells at the same height as the cell measurement was subtracted to remove the quartz glass and PBS signals.

The procedure for extracting autofluorescence and Raman spectral components is illustrated in Fig. S1B and C. To obtain an autofluorescence spectrum, the difference spectra were calculated by subtracting the last spectrum (60–90 s) from the first spectrum (0–30 s) recorded in the time series (Fig. S1B). The difference spectrum consists exclusively of photobleaching components correlated to electronic transitions, such as fluorescence and resonance Raman scattering. On the assumption that the photobleaching low-frequency component of the difference spectrum is derived from autofluorescence, a baseline of the difference spectrum was calculated and considered the autofluorescence spectral component. The baseline curve was fitted using an automatic baseline fitting function based on the least-squares polynomial curve fitting implemented in the Baseline Fitting package (ver. 4) of Igor 9.0 software. The basic algorithm is similar to that described by Lieber and Mahadevan-Jansen (42). A polynomial order of 12 and a presmoothing factor of 20 were used. This function was fitted iteratively. The data points of the fitted curve spectrum that have a higher intensity than the values at the corresponding pixels of the input spectrum were replaced by the intensity of the input data, thereby truncating the data points representing the Raman peaks. The output-fitted spectrum is used as the input for the next iteration.

Due to the minimal impact of photobleaching on the resonance Raman peaks observed for some bacterial strains, the resonance Raman spectra of the carotenoids were extracted from the measured time series data by subtracting the baseline representing the autofluorescence component from the initial spectrum (0–30 s) (Fig. S1C). With the exception of *A. vinosum*, the baseline was calculated using the same functions and parameters as in Fig. S1B. For *A. vinosum*, the polynomial approximation was unable to perfectly fit its baseline, exhibiting a high curvature in the proximity of the carotenoid peaks. Consequently, the Arc Hull function was used to remove autofluorescence components. The signal-to-noise ratio (SNR) of the resonance Raman peaks of carotenoids was calculated by dividing the mean intensity of 3–7 points surrounding the resonance Raman peak (signal) by the root mean square of the intensities in the silent region (2,204–2,274 cm^−1^, 50 points) (noise). In the case of environmental samples, the maximum intensity point around the resonance Raman peak was used for signal intensity. The Raman spectra containing carotenoid peaks with SNR <2.0 for model phototrophic bacteria and <2.5 for environmental samples were excluded in the subsequent analysis.

The analysis of the resonance Raman spectra was as follows. The Raman peaks of the C = C and C–C stretching modes were fitted with a single Lorentzian function to determine their peak positions (wavenumber). All resonance Raman spectra of the model bacterial species were normalized to the intensity of the C = C stretching peak at ~1,500 cm^−1^, and the average Raman spectrum of each species was calculated. To calculate the intensity of the carotenoid Raman peaks in the model bacterial species, the average intensity of 3–7 points around the resonance Raman peaks was normalized to the vector norm of the C–H stretching region of the last spectrum recorded in the time series (smoothed using a 15-point Savitzky–Golay polynomial ﬁlter of a polynomial order of 2).

The analysis of the autofluorescence spectral component was as follows. To calculate autofluorescence intensity, the autofluorescence spectral component was normalized to the vector norm of the C–H stretching region above. Autofluorescence intensity was defined as the maximum intensity at 737–776 nm. The threshold for determining whether the cells collected from clover leaves showed autofluorescence was set to the highest autofluorescence intensity of nonphototrophic *B. subtilis* (*n* = 25), measured on a quartz coverslip. Principal component analysis (PCA) and X-means clustering were performed on the autofluorescence spectra (665–775 nm), truncating divergence at both ends of the spectrum caused by the fitting, followed by minimum–maximum normalization, which rescales data values to a range from 0 to 1. The X-means clustering algorithm was used to classify the autofluorescence spectra of the cells from the clover sample according to similarity without the need to assume the number of clusters. X-means clustering was performed using seven principal components with the following parameters: the type of splitting creation (criterion) was the Bayesian information criterion, and the stop condition for each iteration (tolerance) was 0.0001. PCA and X-means clustering were performed using the scikit-learn (1.0.2) and PyClustering packages (0.10.1.2) in Python (3.7.1), respectively (43, 44).

### Analysis of the 16S rRNA gene sequences

The 16S rRNA genes of the isolates were amplified from fresh colonies on PYS agar using the 27F (5ʹ-AGAGTTTGATCCTGGCTCAG-3ʹ) and 1492R (5ʹ-GGTTACCTTGTTACGACTT-3ʹ) primers (45, 46) with KOD One PCR Master Mix (Toyobo, Osaka, Japan). PCR amplification was performed using a LifeECO Thermal Cycler (Hangzhou Bioer Technology Co., Ltd., Hangzhou, China) under the following conditions: initial denaturation at 94°C for 3 min; 25–30 cycles of denaturation at 98°C for 10 s, primer annealing at 55°C for 5 s, and extension at 68°C for 10 s; and final elongation at 68°C for 10 min. Sequencing of PCR products using the 27F primer was performed by Eurofins Genomics (Tokyo, Japan), and the homology of the sequence (790–810 bp) was compared using the National Center for Biotechnology Information (NCBI) BLAST (NCBI, USA) restricted to type material.

## RESULTS

### Observed single-cell spectra from different strains of the model phototrophic and nonphototrophic bacteria

**Fig 1:**
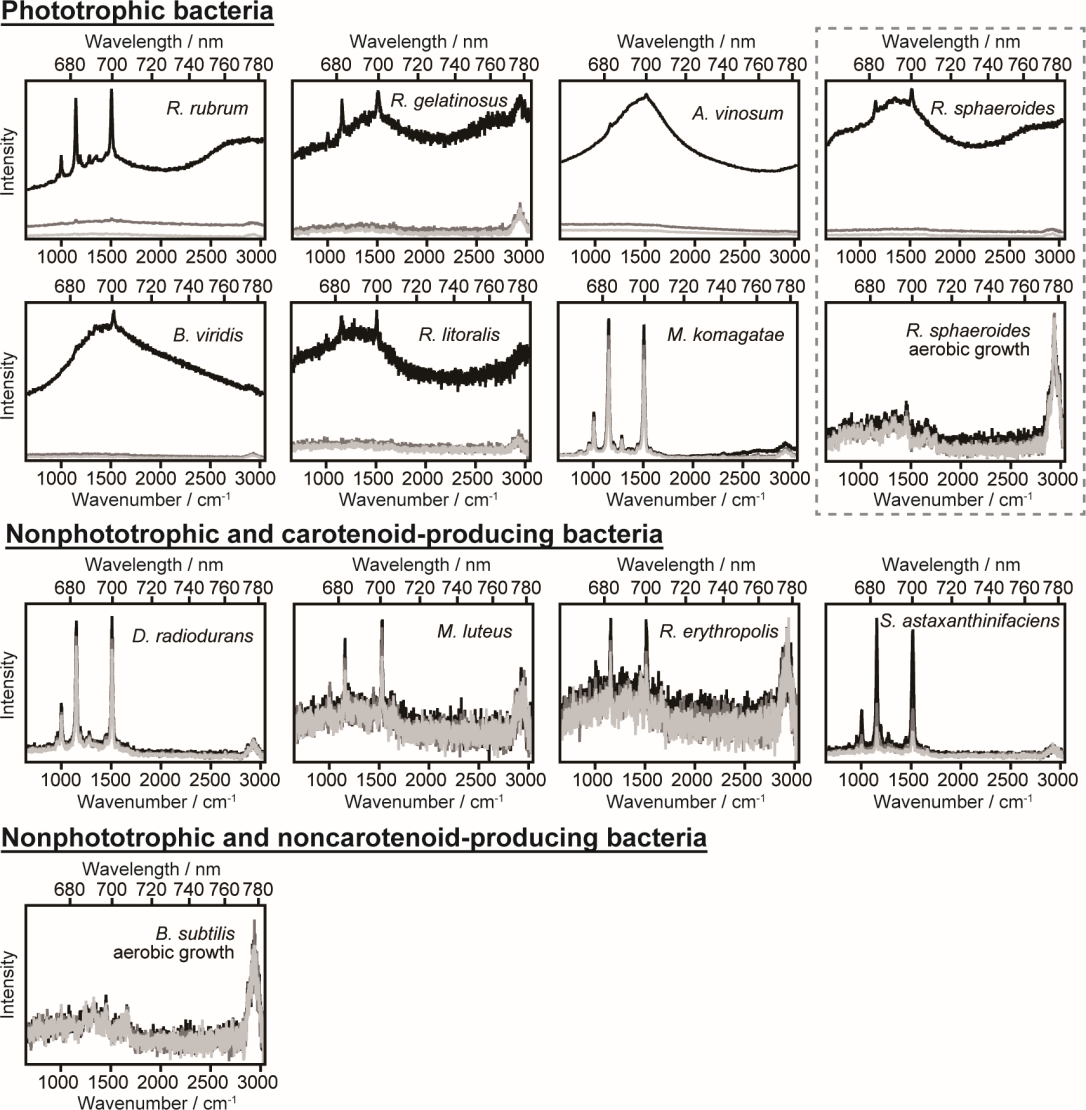
Representative single-cell spectra of seven phototrophic bacteria, four nonphototrophic and carotenoid-producing bacteria, and a nonphototrophic and noncarotenoid-producing bacterium. The two graphs enclosed by gray-dotted lines represent the spectra of a cell of *R. sphaeroides* cultured under phototrophic and aerobic growth conditions, respectively. The single-cell spectra were measured every 30 s up to 90 s (0–30 s, black; 30–60 s, gray; and 60–90 s, light gray lines).

Figure 1 shows the representative single-cell spectra of seven phototrophic bacteria, four nonphototrophic and carotenoid-producing bacteria, and one nonphototrophic and noncarotenoid-producing bacterium. Three sequentially recorded spectra (see Materials and Methods and Fig. S1A) are displayed. In the phototrophic bacterial strains cultured under conditions that induced photosynthetic pigment synthesis, autofluorescence and resonance Raman peaks of carotenoids appeared in the first (0–30 s) spectrum, whereas in the second (30–60 s) and third (60–90 s) spectra, these signals were drastically reduced due to photobleaching. The first spectrum consisted of two resonance Raman peaks of carotenoids at 1,150 and 1,500 cm^−1^ on top of a slowly varying spectrum derived from autofluorescence. In *R. sphaeroides* cultured under aerobic conditions, where photosynthetic pigment synthesis is suppressed, the pigment spectra were barely detectable. The single-cell spectra of the nonphototrophic and carotenoid-producing bacteria exhibited the two characteristic peaks of carotenoids as in phototrophic bacteria, but no pronounced autofluorescence spectrum components were identified.

### Characteristics of the resonance Raman spectra of carotenoids obtained from phototrophic and carotenoid-producing bacteria

**Fig 2:**
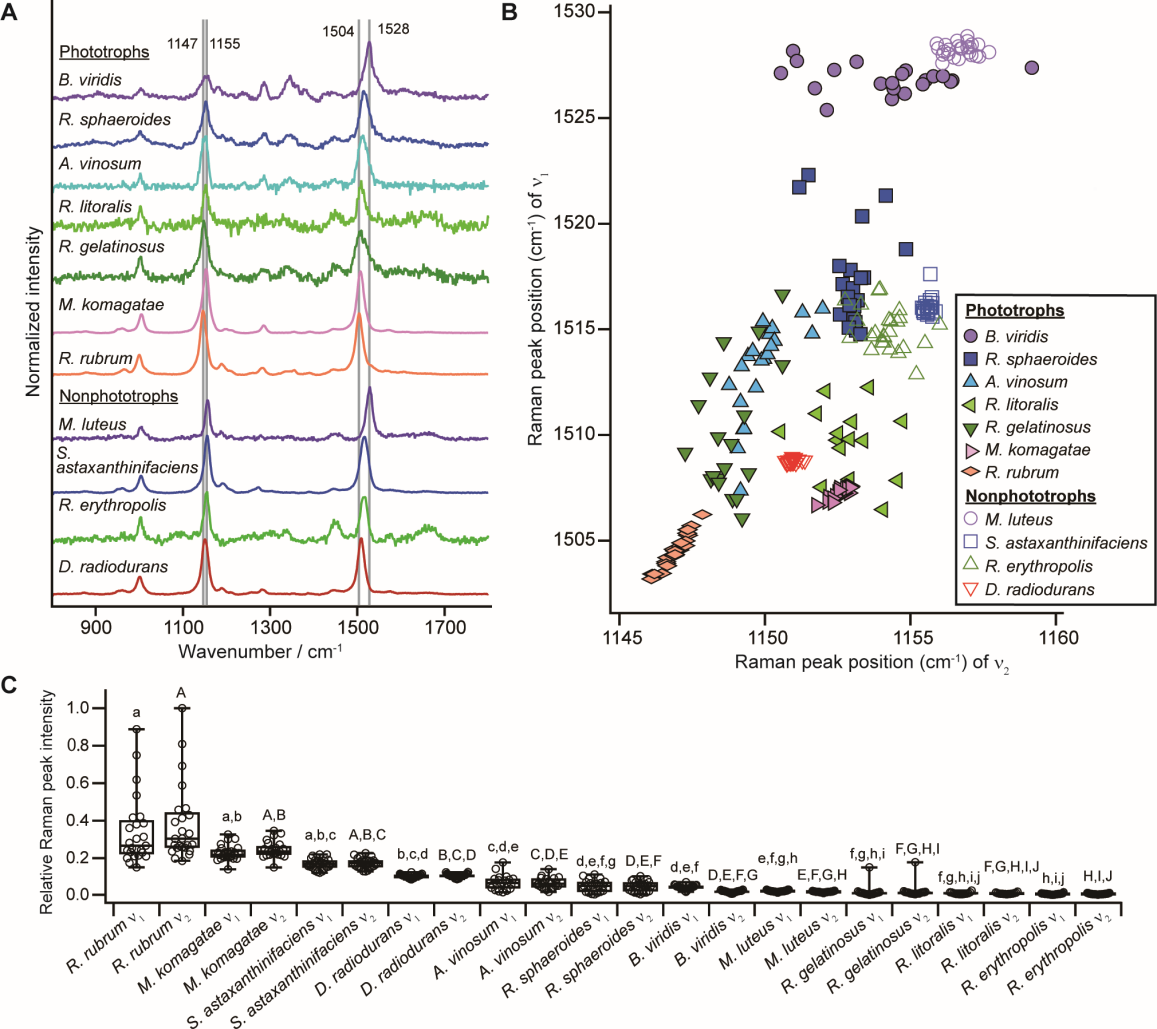
(A) Averaged Raman spectra of the seven phototrophic bacteria and four nonphototrophic and carotenoid-producing bacteria. (B) C = C stretching (ν_1_) peak position vs. C–C stretching (ν_2_) peak position of carotenoids detected in single-cell Raman spectra of the same species as in A. Of the 25 cells, cells with resonance Raman peaks of carotenoids are shown: 21 cells for *B. viridis*, 23 for *R. sphaeroides*, 19 for *R. gelatinosus*, 15 for *R. litoralis*, and 25 for each of the other species. (C) Box plot of the Raman peak intensity for each cell of the 11 bacterial species. The Raman intensity is relative to that of the strongest ν_2_ peak of *R. rubrum*. A significance test was performed for each of the ν_1_ and ν_2_ peaks. Different letters indicate statistically significant differences (*P* < 0.05) among the 11 species using a nonparametric Kruskal–Wallis test followed by a post hoc Dunn–Holland–Wolfe multiple comparison test.

Figure 2A shows the averaged resonance Raman spectra of the carotenoids obtained with the procedures described in the preceding section (Fig. S1C). The resonance Raman peaks at ~1,500 and 1,150 cm^−1^ are assigned to the C = C stretching (ν_1_ peak) and C–C stretching (ν_2_ peak) vibrational modes of the conjugated chain in carotenoids, respectively. Among the phototrophic bacteria, the ν_1_ peak position of *B. viridis* was the highest (~1,530 cm^−1^) among the seven phototrophic bacterial species, and *R. rubrum* showed the lowest ν_1_ peak position at ~1,505 cm^−1^. The main carotenoids of *B. viridis*, *R. sphaeroides*, *A. vinosum*, *R. gelatinosus*, *R. litoralis*, *M. komagatae*, and *R. rubrum* in the order of descending wavenumbers are 1,2-dihydroneurosporene (*N* = 9), spheroidene (*N* = 10), rhodopin (*N* = 11), spheroidene series and spirilloxanthin (*N* = 13), spheroidenone (*N* = 10 with one C = O), glucosyl C_30_ carotenoid (*N* = 11 with two C = O) (inferred from the same genus but different species), and spirilloxanthin (*N* = 13) (47–49), respectively. The ν_1_ peak positions observed in this study (see Fig. 2A) agree well with those reported in the literature: phototrophic bacteria containing spirilloxanthin showed the ν_1_ peak at ~1,505 cm^−1^, and *R. sphaeroides* showed the ν_1_ peak at ~1,519 cm^−1^ (50, 51). The correlation between the ν_1_ peak position and the conjugated chain length of carotenoids is well-known (18, 19)—the longer the conjugated chain length, the lower the ν_1_ peak position (52). A similar correlation between the ν_1_ peak position and the length of the conjugated double bond was observed in nonphototrophic and carotenoid-producing bacteria. The primary carotenoids identified in *M. luteus*, *R. erythropolis*, and *D. radiodurans* are reported to be sarcinaxanthin (*N* = 9), 4-keto-γ-carotene [*N* = 11 (one is from the terminal ring) with one C = O], and deinoxanthin [*N* = 12 (one is from the terminal ring) with one C = O], respectively (50, 53–56).

Figure 2B shows the distribution of the peak positions of the ν_1_ and ν_2_ peaks of carotenoids in the cells of the 11 bacterial strains. The distributions of Raman peak positions were similar in *R. gelatinosus* and *A. vinosum*, whereas those of the other strains were roughly separated from each other. According to previous studies (50, 51), *R. gelatinosus* cells contain both spheroidene and spirilloxanthin, and the ν_1_ peak position appears to be an intermediate between the two carotenoids (i.e., ~1,505 and ~1,519 cm^−1^). Raman peaks derived from carotenoids with 11–13 conjugated C = C bonds appear in the 1,510–1,517 cm^−1^ range in the single-cell Raman spectrum of *Allochromatium* species (57), and the results of our study were intermediate within this range. *R. gelatinosus*, *A. vinosum*, and *R. litoralis* cells were difficult to distinguish by looking only at the distribution of the ν_1_ peak position, but *R. litoralis* could be distinguished using the ν_2_ peak position, which was higher in *R. litoralis* than in the other two strains. Spheroidenone, the main carotenoid of *R. litoralis,* is a ketolated derivative of spheroidene in which a C = O bond is added to the C = C bond framework. The ν_1_ peak position of spheroidenone in light-harvesting complex 2 is shifted to the lower wavenumber side by 4 cm^−1^ relative to that of spheroidene (22). Consistent with this observation, the ν_1_ peak positions of the carotenoids of *R. litoralis* cells were distributed on the lower wavenumber side than those of *R. sphaeroides* cells containing spheroidene. The ν_1_ and ν_2_ peak positions of *M. komagatae* differed from those of other phototrophic bacteria. Several *Methylobacterium* species contain glucosyl C_30_ carotenoids (*N* = 11 with two C = O bonds) and spirilloxanthin (47, 48). These C_30_ carotenoids with keto groups probably had a major influence on the peak positions of *M. komagatae*. In addition to the keto group, the conjugated terminal ring affects the position of the ν_1_ peak, but the effect is regarded as being less pronounced than that of an additional conjugated length (21). These factors may have influenced the distribution of the peak positions in nonphototrophic bacteria, with the exception of *M. luteus. S. astaxanthinifaciens* contains an unidentified primary carotenoid and astaxanthin [*N* = 11 (two from terminal rings) with two C = O] as the second major carotenoid (53). The Raman peak positions of *S. astaxanthinifaciens* are separated from those of astaxanthin (1,520 and 1,157 cm^−1^) in algal cells (58), and it is hypothesized that the Raman peak positions of *S. astaxanthinifaciens* are influenced by the unidentified carotenoid.

There is considerable variation in the carotenoid peak positions across bacterial species, such as *R. gelatinosus*. This large intraspecies variation could be attributed to the carotenoid peaks with a low SNR. In fact, the intensities of the carotenoid Raman peaks varied greatly between species (Fig. 2C). However, in species such as *M. luteus*, carotenoid peaks had low SNR but little variation (Fig. S2). When multiple carotenoids are present in a cell, the carotenoid peaks may become broad and asymmetric. In fact, in *R. gelatinosus*, the full width at half maximum of the ν_1_ peak of the average spectrum was 29.9 cm^−1^. By contrast, the value observed in *M. luteus* was 16.7 cm^−1^. The compositional heterogeneity of different carotenoids within individual cells of species that produce multiple carotenoids may therefore account for the large cell-to-cell variation in carotenoid peak positions.

### Characteristics of the autofluorescence spectra obtained from phototrophic bacteria

**Fig 3:**
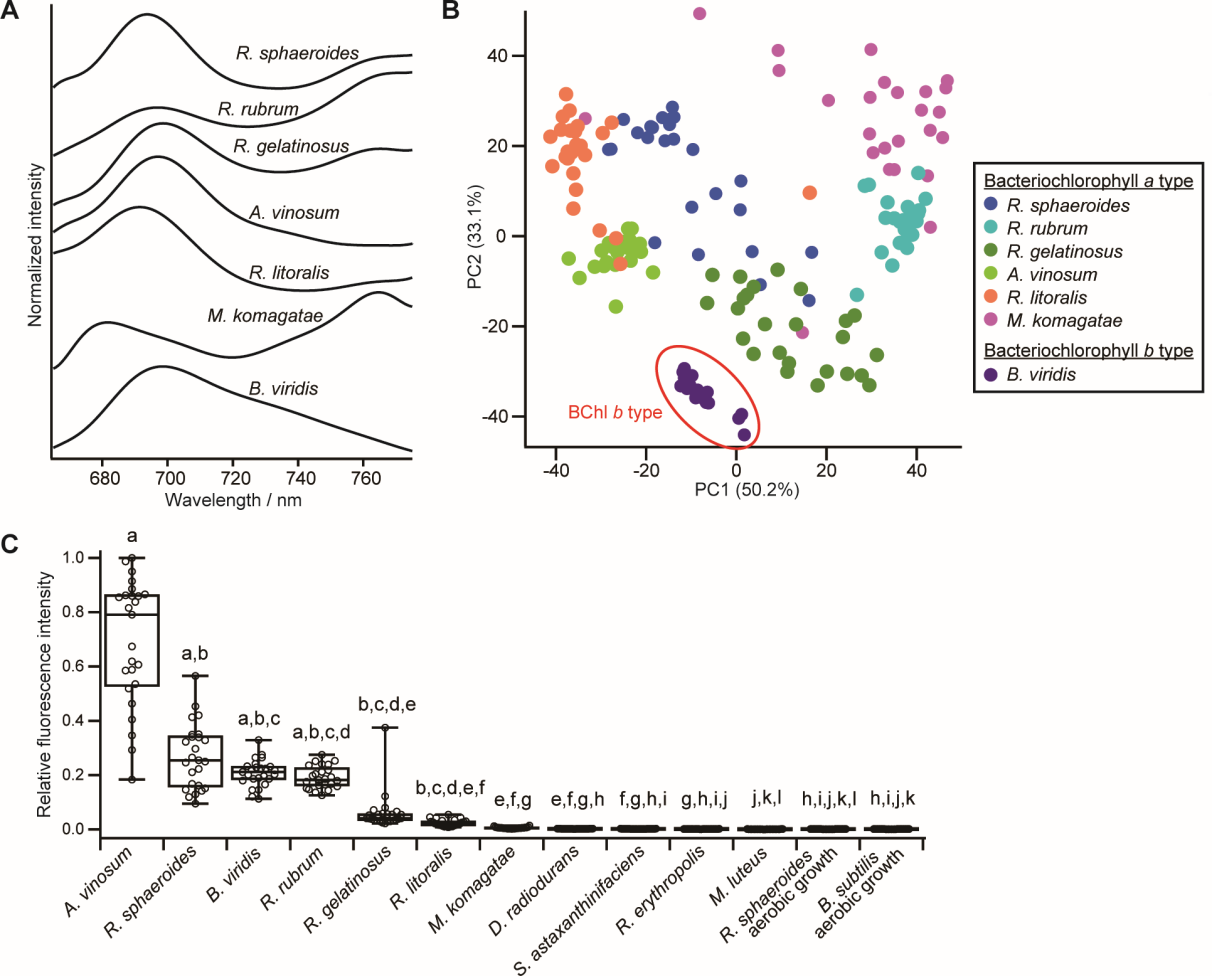
(A) Averaged autofluorescence spectra of the seven phototrophic bacteria. The spectra in the graph, from top to bottom, are those of four types of anaerobic phototrophic bacteria, two types of aerobic phototrophic bacteria that produce BChl *a*, and one type of anaerobic phototrophic bacteria that produce BChl *b*. (B) PCA score plot (PC1 vs. PC2) based on the autofluorescence spectra of cells from model phototrophic bacteria. (C) Box plot of autofluorescence intensity for each cell of the 11 bacterial species (for *R. sphaeroides*, both anaerobic and aerobic growth). Autofluorescence intensity is relative to that of the strongest autofluorescence of *A. vinosum*. Different letters indicate statistically significant differences (*P* < 0.05) among the 12 samples by a nonparametric Kruskal–Wallis test followed by a post hoc Dunn–Holland–Wolfe multiple comparison test.

The averaged autofluorescence spectra of the five anaerobic and two aerobic anoxygenic phototrophic bacteria are shown in Fig. 3A. The fluorescence peak at approximately 690 nm was common to all anaerobic phototrophic bacteria. In *R. sphaeroides, R. rubrum, R. gelatinosus*, and *A. vinosum*, which have BChl *a*, the fluorescence intensity increased from ~740 nm to longer wavelengths, suggesting the presence of one or more fluorescence peaks outside the measurement range. A shoulder peak at ~760 nm was detected in these strains excluding *A. vinosum*. For *B. viridis* with BChl *b*, neither an increase in fluorescence intensity toward 800 nm nor a shoulder at ~760 nm was observed, although a slight bulge was observed at ~740 nm. In the case of the two strains of aerobic phototrophic bacteria, the shape of the *R. litoralis* spectrum was similar to that of the anaerobic phototrophic bacteria. The autofluorescence spectrum of *M. komagatae*, although much weaker in intensity compared with the other species, exhibited a different shape with apparent peaks at ~680 and ~760 nm but showed an increasing trend toward longer wavelengths similar to all other species, excluding *B. viridis*.

Figure 3B shows the PCA scatter plot of the autofluorescence spectra of all phototrophic bacterial cells. Despite the similarities described in the overall shape of the autofluorescence spectra across phototrophic bacterial species, the PCA results demonstrated that they are roughly clustered by species. Notably, *B. viridis*, which contains BChl *b*, formed a well-separated cluster from the other strains, which contained BChl *a*. Consistently, in hierarchical clustering analysis, the autofluorescence spectra of *B. viridis* were grouped into a single cluster (Fig. S3A).

Figure 3C compares the distribution of autofluorescence intensity among phototrophic and nonphototrophic bacteria. Compared with nonphototrophic bacteria, the autofluorescence intensity of phototrophic *A. vinosum, R. sphaeroides, B. viridis*, and *R. rubrum* was significantly higher. Among phototrophic bacteria, the autofluorescence intensity of *R. gelatinosus* and the aerobic phototrophic bacteria *R. litoralis* and *M. komagatae* was low. The reason for this is likely due to the small amount of pigment per cell.

### Raman and autofluorescence spectra of phyllosphere microbial cells from the leaf surface

**Fig 4:**
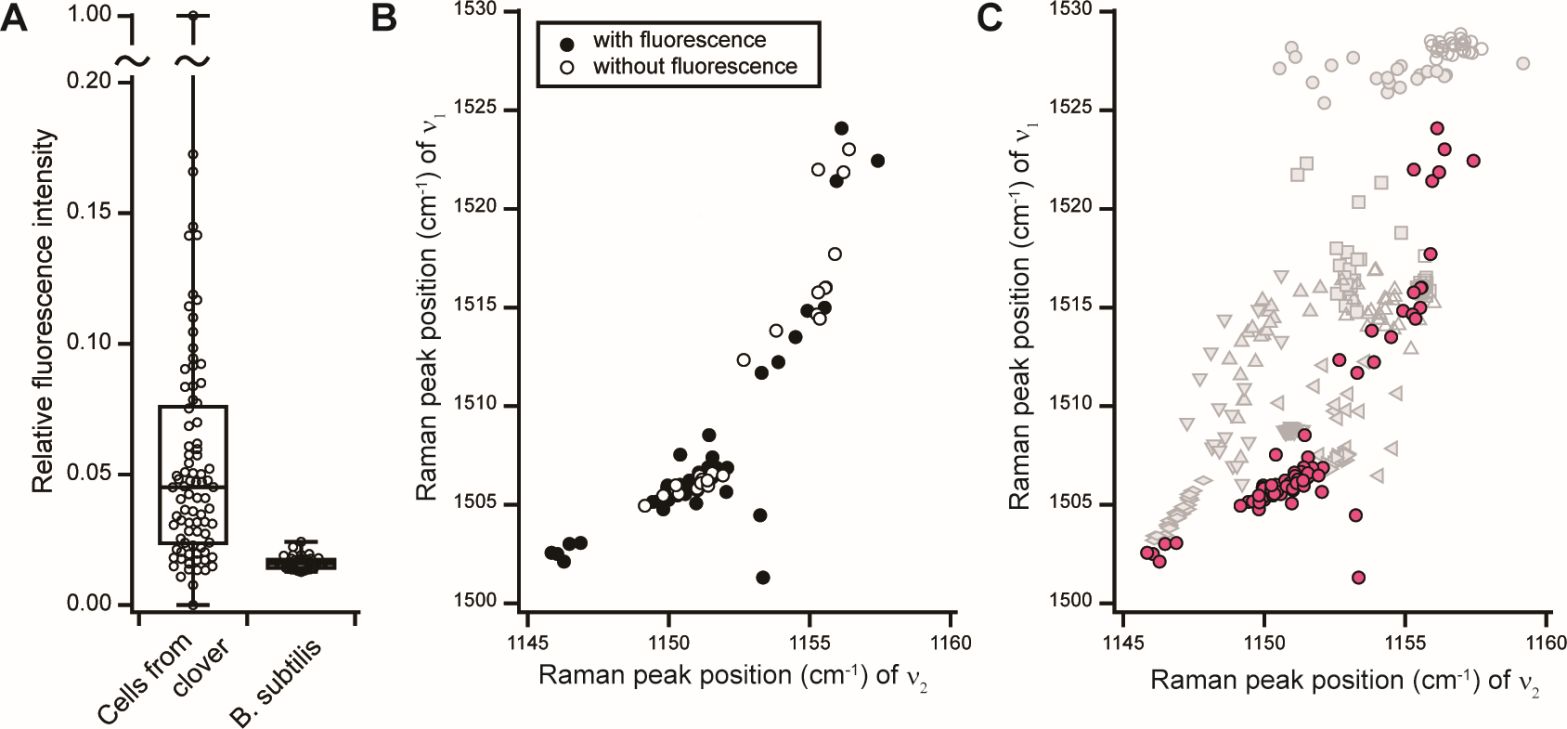
(A) Box plot of autofluorescence intensity for each cell collected from clover leaves that showed carotenoid Raman peaks and for cells of *B. subtilis* as a nonphototroph. Autofluorescence intensity is relative to that of the strongest autofluorescence of a cell from clover leaves. (B) C = C stretching (ν_1_) peak position vs. C–C stretching (ν_2_) peak position of carotenoids in each cell collected from clover leaves. Of 85 cells, 23 cells (open circles) showed resonance Raman peaks of carotenoids without autofluorescence, whereas 62 cells (closed circles) showed carotenoid Raman peaks with autofluorescence. (C) The scatter plot depicts the ν_1_ vs. ν_2_ peak position of carotenoids of the model bacteria (Fig. 2B, gray symbols) overlaid with that of the clover sample (Fig. 4B, red circles).

Of the 100 cell-like particles (<5 µm) obtained from the plant leaf sample, we detected typical resonance Raman peaks of carotenoids in 85 particles. Although we cannot rule out the possibility that these particles were fragments of plant origin, such as pollen, the detection of various carotenoids corroborates that they were prokaryotic cells and not nonbiological objects. Several spectra in which carotenoid peaks were detected showed an autofluorescence-derived baseline (Fig. S4) similar to those of phototrophic bacteria (Fig. 3A). Figure 4A is a box plot of the autofluorescence intensities of the 85 cells. The autofluorescence intensity of the cells obtained from clover was basically low. Data for *B. subtilis* cells are shown for comparison. One cell exhibited a markedly high autofluorescence intensity (corresponding to Fig. S4C), and others showed intensities of <0.18 of that cell. The ν_1_ peak position of the carotenoids varied in the range of 1,500–1,525 cm^−1^ (Fig. 4B). Because the distribution of the ν_1_ peak position for each phototrophic bacterial species studied was <10 cm^−1^ (Fig. 2B), it is likely that >3–4 types of carotenoids were contained in the 85 clover leaf cells. Figure 4C shows the ν_1_ and ν_2_ peak positions of the carotenoids in the clover sample (red circles; same as in Fig. 4B) overlaid with those in model bacteria (gray symbols; same as in Fig. 2B). The distribution of the ν_1_ peak position in the 1,505–1,517 cm^−1^ range was similar to that of the model bacteria containing carotenoids with keto groups and/or terminal rings. The ν_1_ peak positions of the five cells from the clover sample were at ~1,503 cm^−1^, lower than that in *R. rubrum*, which has spirilloxanthin with the longest conjugated length of 13 among the model bacteria studied here. This suggests that these cells possess linear carotenoids with a conjugated length of 13 (e.g., oscillol glycoside) (59) or may possess additional linear carotenoids with a conjugation length of 13 and a keto group (e.g., 2,2'-diketospirilloxanthin) (49). Of the 85 cells, 62 (Fig. 4B, closed circles) showed autofluorescence above a threshold intensity (see Materials and Methods for definition of threshold), but the positions of the Raman peaks of carotenoids they displayed were not different between cells with and without autofluorescence.

**Fig 5:**
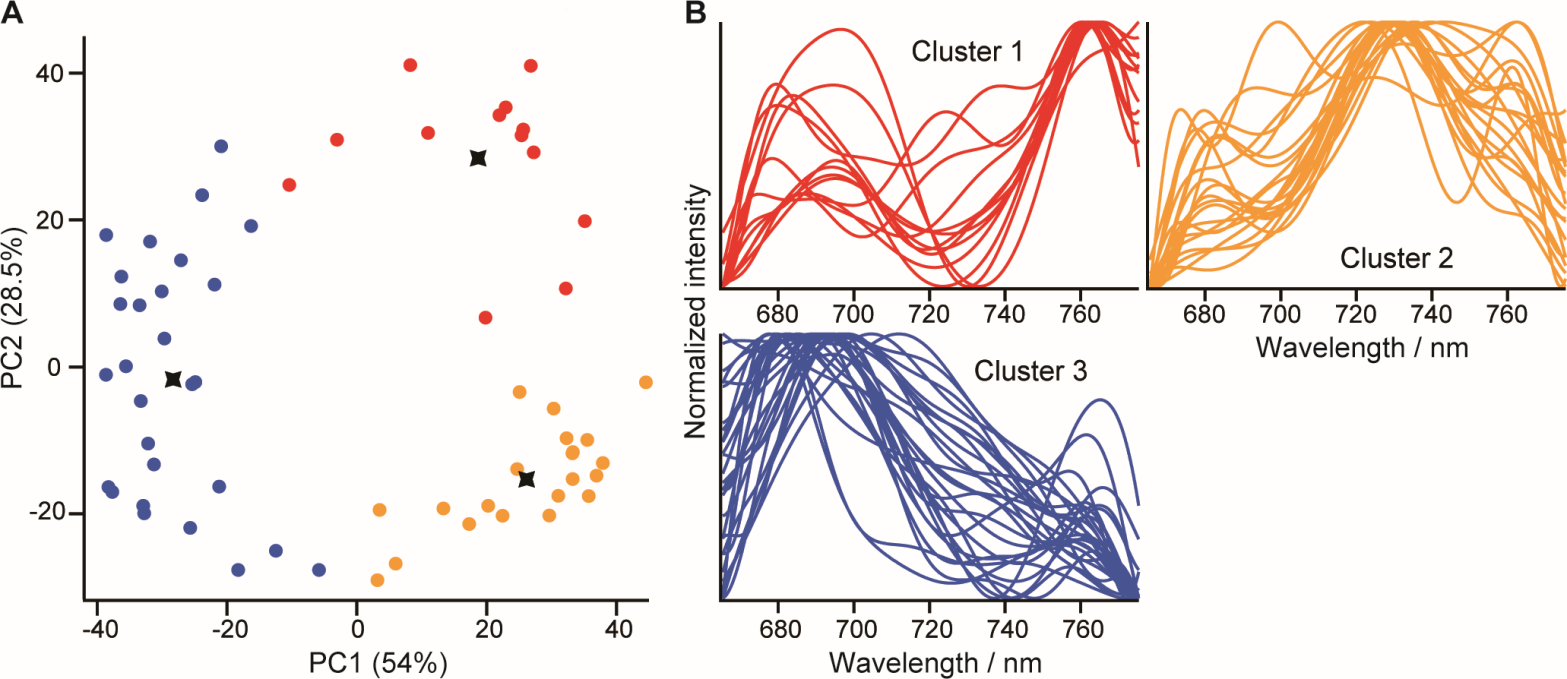
(A) PCA score plot (PC1 vs. PC2) and X-means clustering based on the autofluorescence spectra of cells from clover leaves showing autofluorescence intensity above a threshold value. Different colors indicate clusters 1–3 (1, red; 2, yellow; and 3, blue), and cross marks indicate cluster centroids. (B) Autofluorescence spectra of individual cells in clusters 1–3.

PCA and X-means clustering were performed to classify autofluorescence spectra by spectral shape (Fig. 5). Autofluorescence spectra were divided into three clusters. Despite large cell-to-cell variations, it appears that the spectra of clusters 1 and 3 had major peaks at 680–700 nm and ~760 nm. The spectral shapes of these clusters were somewhat similar to those of model phototrophic bacteria. Cluster 2 had a peak near 730 nm—a shape that was different from all phototrophic bacteria studied.

### Phylogenetic and spectroscopic characteristics of the strains isolated from the phyllosphere

Six microbial strains with pink, orange, and yellow colonies were isolated (STP2, STP3, STO1, and STY1-3, respectively). Phylogenetic analyses based on the 16S rRNA gene revealed that these strains are closely related to the genera *Methylobacterium*, *Sphingomonas*, *Curtobacterium*, and *Chryseobacterium* (Table 1). These microbes have been detected in the aboveground parts of plants (14, 60–65). Among these microbes, species containing BChl have been reported for the genera *Methylobacterium* (66) and *Sphingomonas* (67). *Methylobacterium* has been well studied for pigment production and genomic information (26, 47, 48, 66, 68, 69). Figure S5A and C show the absorption spectra of the pigments extracted with methanol from the colonies of strains STP2 and STP3. Peaks between 400 and 550 nm are assigned to carotenoids and an additional small peak at 770 nm to BChl *a*. Cells of strains STP2 and STP3 showed the ν_1_ resonance Raman peak of carotenoids at 1,506–1,507 cm^−1^ (Fig. S5; Table 1) and autofluorescence spectrum similar to those shown in Fig. S4A, suggesting that this autofluorescence spectrum is associated with intracellular pigments, such as BChl *a* analogs of *Methylobacterium*.

The ν_1_ and ν_2_ peak positions of STY1 and STY2 (1,522–1,523 cm^−1^ for ν_1_ and 1,156–1,157 cm^−1^ for ν_2_; see Table 1) were similar to those of microbes with β-carotene [*N* = 11 (two from the terminal ring)], as reported in other studies (70). β-carotene and carotenoids with similar structures have been found in the genera *Sphingomonas* and *Curtobacterium* (71, 72), which are closely related to the isolated strain. The cells observed in the 1,520–1,525 cm^−1^ regions of the scatter plot in Fig. 4B and C (carotenoid peaks of the model microbes used in this study did not fall in this range) are considered microbes containing β-carotene or carotenoids with similar structures. The Raman peak positions of STY3 (1,531 cm^−1^ for ν_1_ and 1,133 cm^−1^ for ν_2_) were markedly different from those of the other isolated strains and model microbes but resembled those of flexirubin (73), a polyene compound structurally different from carotenoids. Flexirubin-type pigments are produced by bacteria in several genera of the phylum *Bacteroidota*, including the genus *Chryseobacterium*, which is closely related to STY3 (73, 74).

## DISCUSSION

In this study, we have demonstrated the simultaneous detection of the resonance Raman and autofluorescence spectra of pigments in purple phototrophic bacteria and microbes on the surface of plant leaves at the single-cell level. Conventional methods to detect phototrophic bacterial cells have relied on the use of BChl *a* fluorescence signals through the use of infrared epifluorescence microscopy. In comparison, our method could simultaneously detect the resonance Raman spectra of different types of carotenoids and autofluorescence spectra from cells containing BChls *a* and *b*. Recently, the autofluorescence and Raman spectra of BChl *a* from cells of aerobic phototrophic bacteria were acquired using 785 nm excitation, but no carotenoid signatures were detected because the excitation wavelength was too far from the absorption band of carotenoids to achieve sufficient resonance enhancement and because the strong fluorescence of BChls interferes with Raman signals (75). The advantage of our approach using 632.8 nm excitation is the ability to detect both multiple BChls and carotenoids with reasonable clarity due to the relatively weak autofluorescence in the detection wavelength region.

### Origin and nature of the autofluorescence spectra of model phototrophic bacteria

The fluorescence peaks detected were observed in the range of 660–780 nm (Fig. 3A). In BChl *a*-containing phototrophic bacteria, the Q_y_ absorption peak appears at >800 nm, and the fluorescence peak appears at 880–890 nm. Therefore, the origin of the fluorescence peaks detected is not BChl *a* itself but some pigment(s) associated with BChl *a*. Studies have reported weak fluorescence peaks around this region from purified light-harvesting complex 1 and 2, which were attributed to the photo-oxidation products of BChl *a* during protein purification (76, 77). The fluorescence peak observed in this region may have been caused by photo-oxidation products originally present in the cells or by laser irradiation. Similarly, the autofluorescence spectrum of the BChl *b*-containing bacterium *B. viridis* may be due to a different form of BChl *b*. Note that although the origin of the autofluorescence spectra detected in this study has not been fully elucidated, these spectral patterns will enable us to detect phototrophic bacteria, including those with BChl *b*, which are usually difficult to detect, and to identify the type of BChls. The excitation wavelength of 632.8 nm falls in the absorption band of chlorophyll *a*. Using our approach in combination with information on environmental conditions, genome, and cell morphology, it would be possible to detect cyanobacteria containing chlorophyll *a* and distinguish them from phototrophic bacteria based on autofluorescence intensity because they show stronger fluorescence than phototrophic bacteria.

The autofluorescence intensity of anaerobic phototrophic bacteria exhibited interspecies variation, with *R. gelatinosus* displaying the lowest autofluorescence intensity (Fig. 3C). Many anaerobic phototrophic bacteria develop an intracytoplasmic membrane system containing a photosynthetic apparatus. However, *R. gelatinosus* is unable to develop such a system to a notable extent (78). We hypothesize that this low level of photosynthetic apparatus development resulted in the observed low autofluorescence intensity in *R. gelatinosus*. The aerobic phototrophic bacteria *R. litoralis* and *M. komagatae* had weak autofluorescence intensity. Consistently, aerobic phototrophs show naturally low expression of the photosynthetic apparatus. For all phototrophic bacteria used in this study, the coefficient of variation of autofluorescence intensity within the species was >20%. It is unclear whether the cell-to-cell variation in autofluorescence intensity reflects the expression of the photosynthetic apparatus or is simply due to technical issues (e.g., variation in experimental conditions). However, if the stability of spectroscopic measurement is improved, cell-to-cell variation can be evaluated quantitatively.

### Variation in carotenoid Raman peaks across bacterial species and cells

Only two of 11 species studied (*A. vinosum* and *R. gelatinosus*) exhibited a substantial overlap in the distribution of the carotenoid peak positions, and the remaining species showed relatively good separation (Fig. 2B). *A. vinosum* and *R. gelatinosus* produce several types of linear carotenoids without keto groups or terminal rings and with different conjugated chain lengths in the range of 10–13 (Table S1), thus making their discrimination difficult. By contrast, it was easier to discern the distribution of the Raman peaks of cells containing carotenoids that differed in conjugation length and whether they had terminal rings or keto groups. The overall trend of hierarchical clustering results (Fig. S3B) was more or less similar to that of the scatter plot (Fig. 2B), but a clearer separation was obtained between *M. luteus* and *B. viridis* with hierarchical clustering than in the scatter plot because a wider region of spectra can be used to detect their differences in multivariate analysis. This finding suggests that we can investigate the presence of variety within a group of cells with similar carotenoid structures by estimating the type of carotenoid structure of the cells. Such an estimation is possible by extracting the peak positions of the carotenoids and then comparing the overall shape of the spectra using a clustering method. The correlation between microbial taxa and carotenoid types is not consistent, but some trends can be recognized in the biosynthesis pathway and evolution (49, 79). For example, many purple phototrophic bacteria (belonging to the phylum *Pseudomonadota*) are known to produce only linear carotenoids such as spirilloxanthin, and the phylogenetic relationships of the core proteins involved in carotenoid biosynthesis are also observed to be grouped together. The expression of pigments in the same microbes can vary depending on the culture conditions and growth stage (49, 80). Our analysis method can be used to detect differences in the types and physiological states of pigmented microbes nondestructively and highly sensitively. An important issue in this regard is the minor shifts in the carotenoid Raman bands that appear to be unrelated to the conjugated chain length but are related to, for example, the excitation wavelength (50). A wavelength-tunable Raman microspectrometer recently developed in our laboratory could be used in future studies to address this issue.

Although the intensity of the resonance Raman peak varied across the bacterial species studied (Fig. 2C), it is considered to be a less robust probe for the discrimination of species or physiological state for the following reasons. The intensity of the resonance Raman peak increases as the excitation wavelength approaches the absorption maximum of the pigment (e.g., 400–600 nm for carotenoids), whose wavelength is longer for carotenoids with longer conjugation lengths (i.e., the more delocalization of the π-electron system). In fact, *R. rubrum* and *M. komagatae*, which possess carotenoids with long conjugation lengths and absorption closer to the excitation wavelength of 632.8 nm than other carotenoids, exhibited stronger Raman peak intensities. Therefore, the resonance Raman intensity of carotenoids does not simply reflect the concentration of carotenoids in the cell but also depends on enhancement factors.

### Significance of the results of phyllosphere microbes

The estimated terrestrial leaf area where microbes could colonize is ~6.4 × 10^8^ km^2^, and the bacterial population of the leaf surface could be up to 10^26^ cells (81). A metagenomic analysis showed that diverse phylogenetic groups of phototrophic bacteria may inhabit plant leaves (12). To understand the biogeochemical cycles in the atmosphere and around plants, it is important to assess how bacteria use light energy to metabolize on plant leaves. Although a large fraction of the phyllosphere cells showed lower autofluorescence intensity compared with the model phototrophs (Fig. S4A, B, and D), the shape of the autofluorescence spectra appeared to be similar for both. The cell with the highest autofluorescence intensity (i.e., cell C in Fig. S4) showed an autofluorescence spectrum similar to that of *B. viridis* (Fig. 3A), but its peak position was slightly lower than that of *B. viridis*. The Raman peak positions of the carotenoids (1,509 and 1,151 cm^−1^) were also lower than those in *B. viridis* (Fig. 2). This result suggests that cell C is a phototrophic bacterium of a different species from the model phototrophic bacteria used in this study. Based on Fig. S4E and F, this cell may not be a minor species in the phyllosphere. Because our method is nondestructive, it is possible to separate characteristic cells, such as cell C, using single-cell separation techniques. We showed that cells displaying diverse types of autofluorescence spectra on the leaf surfaces are abundant. Increasing the number of reference spectra of aerobic phototrophic bacteria and extending spectral coverage to a longer wavelength range (>800 nm) would provide more detailed information on the correlation between bacteria and light on leaf surfaces.

We isolated six bacterial strains from the phyllosphere and identified two belonging to *Methylobacterium* (Table 1). The shape of the autofluorescence spectra of STP2 and STP3 (Fig. S5B and D) is similar to that of cluster 1 of cells from the phyllosphere (Fig. 5B). Therefore, the cells belonging to cluster 1 and showing the peak position of carotenoids at 1,506–1,507 cm^−1^ were possibly *Methylobacterium* species. *Methylobacterium* is ubiquitous and abundant on the leaf surface. These bacteria could grow to use methanol released by the plant—a byproduct of plant cell wall metabolism (82–84)—and to produce some types of plant hormones (85–87). Therefore, they are expected to be important for plant health and coevolution with plants. Nevertheless, it remains unclear how the utilization of light energy is related to the function and colonization ability of *Methylobacterium*. If further physiological studies on *Methylobacterium* reveal a correlation between the expression state of BChl pigments and the autofluorescence spectra obtained by this method, *Methylobacterium* could be identified *in situ* by the resonance Raman peaks of carotenoids and the ecophysiology derived from the autofluorescence spectrum.

Finally, we envisage that our technique will contribute to a better understanding of the role of phototrophic bacteria, which are widely distributed in nature (on leaf and ocean surfaces, etc.). Improving the technique to study the effects of physiological conditions and phylogenetic lineage differences will enable studies on the ecology of pigment-producing microbes in various environments. Furthermore, the combination of this technique with single-cell separation techniques and/or emerging culture techniques, such as microdevices, will offer a new screening method for useful microbes in many fields, such as health and food industries.

## Data Availability

The partial 16S rRNA gene sequences obtained in this study have been deposited in the DDBJ/EMBL/GenBank nucleotide sequence databases with the following accession numbers: LC801384, LC801385, LC801386, LC801387, LC801388, and LC801389.

## SUPPLEMENTAL MATERIAL

Supplemental material aem.00129-25-s0001.pdf Table S1; Figures S1 to S5.

ASM does not own the copyrights to Supplemental Material that may be linked to, or accessed through, an article. The authors have granted ASM a non-exclusive, world-wide license to publish the Supplemental Material files. Please contact the corresponding author directly for reuse.
