# Supplementary material for: Single-cell pigment analysis of phototrophic and phyllosphere bacteria using simultaneous detection of Raman and autofluorescence spectra
Source: Appl Environ Microbiol. 2025 Apr 10;91(5):e00129-25. doi: 10.1128/aem.00129-25 (PMC12093961; doi:10.1128/aem.00129-25)
Supplement: Supplemental material — Table S1; Figures S1 to S5. [file aem.00129-25-s0001.pdf]

# Supporting Information

# Single-cell pigment analysis of phototrophic and phyllosphere bacteria using simultaneous detection of Raman and autofluorescence spectra

Nanako Kanno[a]# and Shinsuke Shigeto[a]#

[a]Department of Chemistry, School of Science, Kwansei Gakuin University, Sanda, Hyogo, Japan

#Address correspondence to

Nanako Kanno, n.kanno4n3a@gmail.com

Shinsuke Shigeto, shigeto@kwansei.ac.jp

**Table S1**

The phylogenetic and carotenoid characteristics of the bacteria studied.

| Species | Phylum, Class, and Order | Carotenoid Pigments | Ref. No. |
|---|---|---|---|
| **Anaerobic Anoxygenic Phototrophic Bacteria** | | | |
| *Allochromatium vinosum* (bacteriochlorophyll *a*) | *Pseudomonadota, Gammaproteobacteria, Chromatiales* | rhodopin, spirilloxanthin, anhydrorhodovibrin, lycopene, rhodovibrin, 3,4-didehydrorhodopin, OH-spirilloxanthin | 1 |
| *Rhodobacter sphaeroides* (bacteriochlorophyll *a*) | *Pseudomonadota, Alphaproteobacteria, Rhodobacterales* | spheroidene, spheroidenone | 1 |
| *Rhodospirillum rubrum* (bacteriochlorophyll *a*) | *Pseudomonadota, Alphaproteobacteria, Rhodospirillaceae* | spirilloxanthin, anhydrorhodovibrin, rhodovibrin, rhodopin, lycopene | 1 |
| *Rubrivivax gelatinosus* (bacteriochlorophyll *a*) | *Pseudomonadota, Betaproteobacteria, Burkholderiales* | OH-spheroidene, spheroidene, OH- spheroidenone, spirilloxanthin | 1 |
| *Blastochloris viridis* (bacteriochlorophyll *b*) | *Pseudomonadota, Alphaproteobacteria, Hyphomicrobiales* | 1,2-dihydroneurosporene, 1,2-dihydrolycopene, 1,2-dihydro-3,4-dehydrolycopene | 1 |
| **Aerobic Anoxygenic Phototrophic Bacteria** | | | |
| *Methylobacterium komagatae* (bacteriochlorophyll *a*) | *Pseudomonadota, Alphaproteobacteria, Hyphomicrobiales* | glucosyl $C_{30}$ carotenoid, spirilloxanthin (these have been reported from other species belonging to genus *Methylobacterium*) | 1,2,3 |
| *Roseobacter litoralis* (bacteriochlorophyll *a*) | *Pseudomonadota, Alphaproteobacteria, Rhodobacterales* | spheroidenone | 1 |
| **Nonphototrophic and Carotenoid-Producing Bacteria** | | | |
| *Deinococcus radiodurans* | *Deinococcota, Deinococci, Deinococcales* | deinoxanthin | 4 |
| *Micrococcus luteus* | *Actinomycetota, Actinomycetes, Micrococcales* | sarcinaxanthin, sarcinaxanthin monoglucoside, sarcinaxanthin diglucoside | 5,6 |
| *Rhodococcus erythropolis* | *Actinomycetota, Actinomycetes, Mycobacteriales* | 4-keto-γ-carotene, γ-carotene | 7 |
| *Sphingomonas astaxanthinifaciens* | *Pseudomonadota, Alphaproteobacteria, Sphingomonadales* | unidentified carotenoid, astaxanthin | 8 |
| **Nonphototrophic and Noncarotenoid-Producing Bacteria** | | | |
| *Bacillus subtilis* | *Bacillota, Bacilli, Caryophanales* | - | - |

**[References in Table S1]**


1. Takaichi S. 1999. Carotenoids and carotenogenesis in anoxygenic photosynthetic bacteria, p. 39–69. *In* Frank HA, Young AJ, Britton G, Cogdell RJ (ed), *The Photochemistry of Carotenoids*. Springer, Dordrecht.
2. Mo XH, Sun YM, Bi YX, Zhao Y, Yu GH, Tan LL, Yang S. 2023. Characterization of $C_{30}$ carotenoid and identification of its biosynthetic gene cluster in *Methylobacterium extorquens* AM1. *Synthetic and Systems Biotechnology* **8**(3):527-535.
3. Osawa A, Kaseya Y, Koue N, Schrader J, Knief C, Vorholt JA, Sandmann G, Shindo K. 2015. 4-[2-O-11Z-Octadecenoyl-β-glucopyranosyl]-4,4′-diapolycopene-4,4′-dioic acid and 4-[2-O-9Z-hexadecenoyl-β-glucopyranosyl]-4,4′-diapolycopene-4,4′-dioic acid: new $C_{30}$-carotenoids produced by *Methylobacterium*. *Tetrahedron Letters* **56**(21): 2791-2794.
4. Lemee L, Peuchant E, Clerc M, Brunner M, Pfander H. 1997. Deinoxanthin: A new carotenoid isolated from *Deinococcus radiodurans. Tetrahedron* **53**(3): 919-926
5. Jehlička J, Edwards HGM, Osterrothová K, Novotná J, Nedbalová L, Kopecký J, Němec I, Oren A. 2014. Potential and limits of Raman spectroscopy for carotenoid detection in microorganisms: implications for astrobiology. *Philosophical Transactions of the Royal Society A: Mathematical, Physical and Engineering Sciences* **372**: 20140199
6. Netzer R, Stafsnes MH, Andreassen T, Goksøyr A, Bruheim P, Brautaset T. 2010. Biosynthetic pathway for γ-cyclic sarcinaxanthin in *Micrococcus luteus*: heterologous expression and evidence for diverse and multiple catalytic functions of $C_{50}$ carotenoid cyclases. *Journal of Bacteriology* **192**(21): 5688–5699
7. Tao L, Picataggio S, Rouviere PE., and Cheng Q. 2004. Asymmetrically acting lycopene β-cyclases (CrtLm) from non-photosynthetic bacteria. *Molecular Genetics and Genomics* **271**: 180–188
8. Asker D, Beppu T, Ueda K. 2007. *Sphingomonas astaxanthinifaciens* sp. nov., a novel astaxanthin producing bacterium of the family *Sphingomonadaceae* isolated from Misasa, Tottori, Japan. *FEMS Microbiology Letters* **273**(2): 140–148

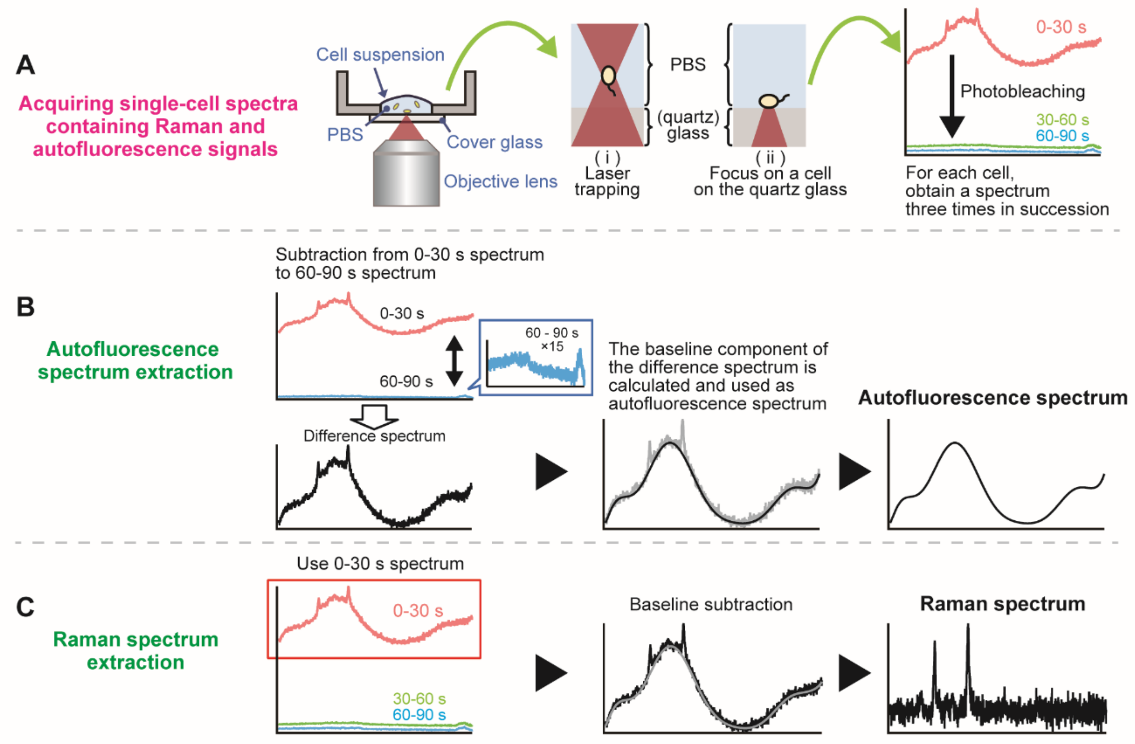


**Figure S1**

Method of (**A**) acquiring single-cell spectra containing Raman and autofluorescence signals and separating into (**B**) autofluorescence and (**C**) Raman spectra. (**A**) A cell suspension was placed in a glass-bottomed dish, and laser light was focused on the suspended cells (in the case of model strains or isolated strains) or on the cells on the quartz glass (in the case of environmental samples, Figures 4, 5, and S4). (i) A cell was captured via the laser trapping technique (also known as optical tweezers), which was used for model or isolated bacterial strains. (ii) When observing environmental samples, the cells attached to the quartz glass were measured to ensure that the shape of the cells was observed prior to Raman measurement. The time-series of Raman spectra of cells was measured every 30 s up to 90 s using 30-s exposure time. As the laser irradiation time increases, the low-frequency autofluorescence curve containing the resonance Raman peaks of the carotenoids decreases drastically in intensity. This decaying behavior is characteristic of laser-induced photobleaching. (**B**) Extraction of the autofluorescence spectrum from the mixed spectrum of the Raman and autofluorescence signals. First, the spectra from the first (0–30 s) to the third (60–90 s) time series were subtracted to obtain the difference spectrum, and the baseline component of the difference spectrum was calculated to obtain the autofluorescence spectrum. (**C**) Extraction of the Raman spectrum from the mixed spectrum of the Raman and autofluorescence signals. The resonance Raman spectrum containing the carotenoid peak was extracted by calculating and subtracting the low-frequency curve derived from the autofluorescence as the baseline from the first spectrum in the time series (0–30 s).

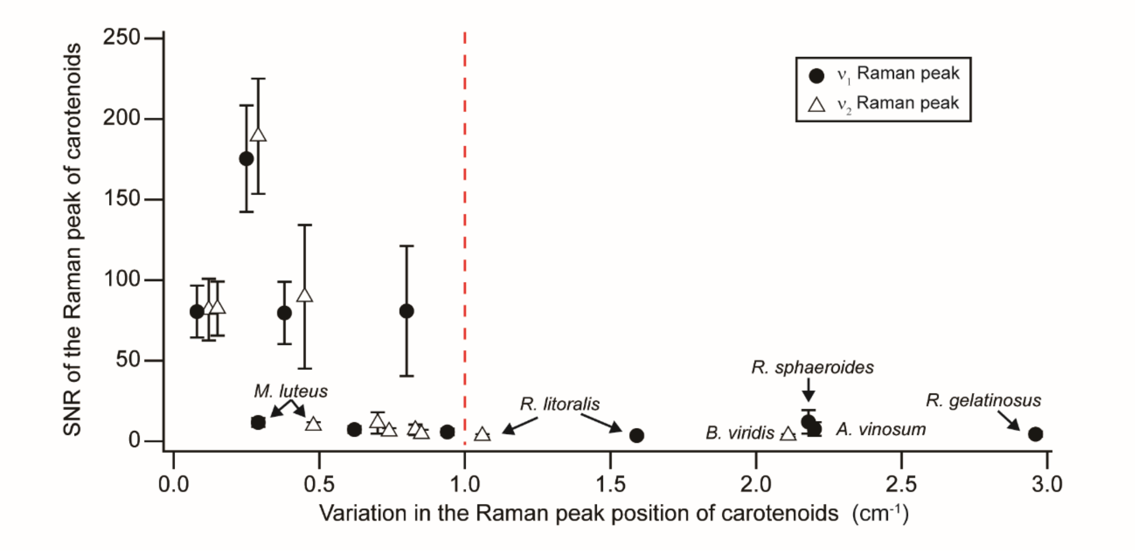


**Figure S2**

Relationship between the signal-to-noise ratio (SNR) of the resonance Raman peaks and the variation in the resonance Raman peak position of carotenoids between cells. Filled circles and open triangles show the resonance Raman peak results for each model species, $\nu_1$ and $\nu_2$, respectively. Red dotted line indicates the boundary where peak variation is 1 $cm^{-1}$. SD was calculated and was set as the variation of the Raman peaks.

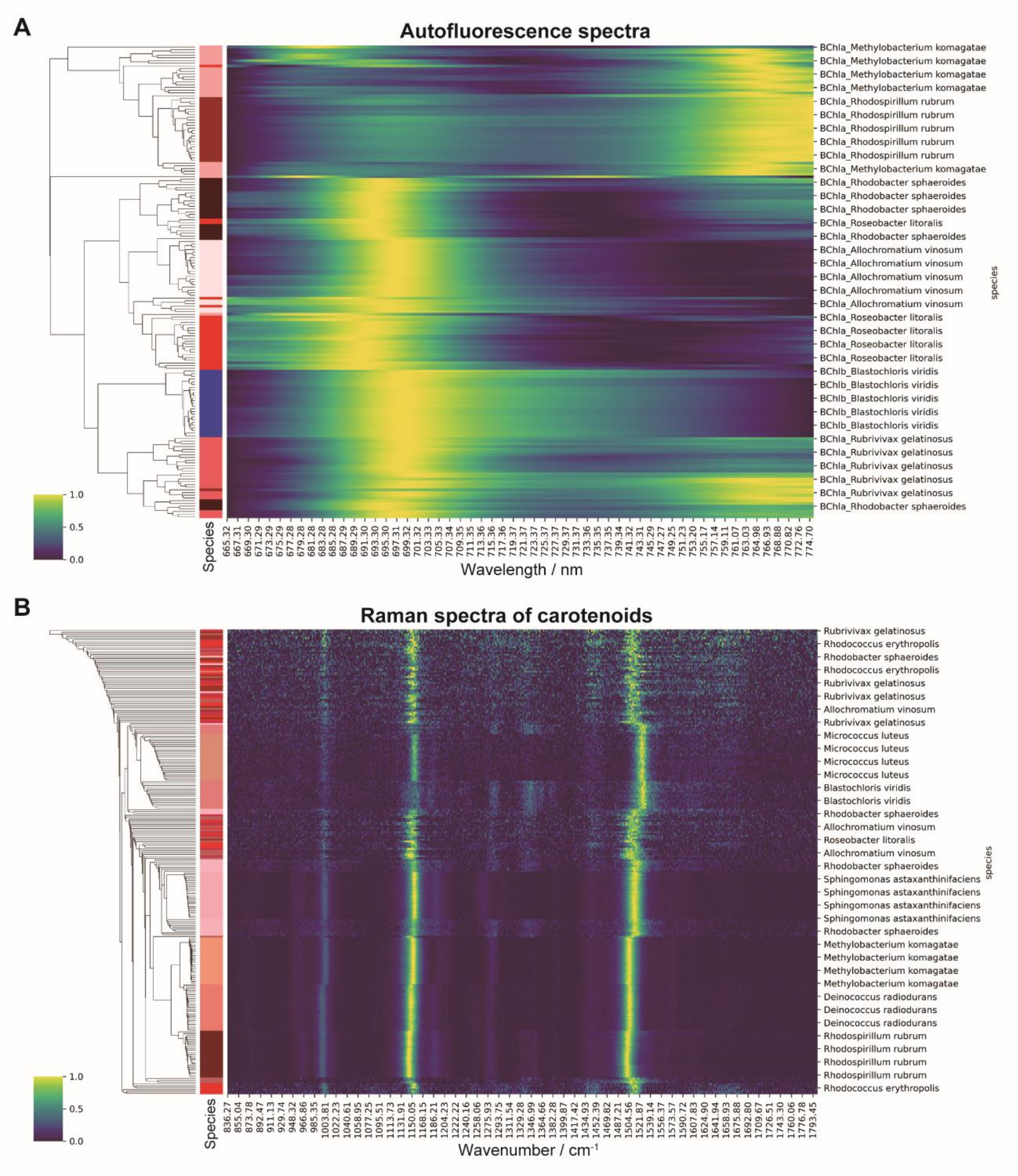


**Figure S3**

Hierarchically clustered heatmap of (**A**) autofluorescence spectra among model phototrophic bacteria and (**B**) resonance Raman spectra among model phototrophic bacteria and nonphototrophic and carotenoid-producing bacteria. (**A**) The same autofluorescence spectrum as that used in principal component analysis in Fig. 3B was used. (**B**) Created using the 836–1,800 $cm^{-1}$ range of the Min–Max normalized resonance Raman spectrum. Hierarchically clustered heatmaps were created by using the seaborne (0.12.2) package in Python (3.11.7).

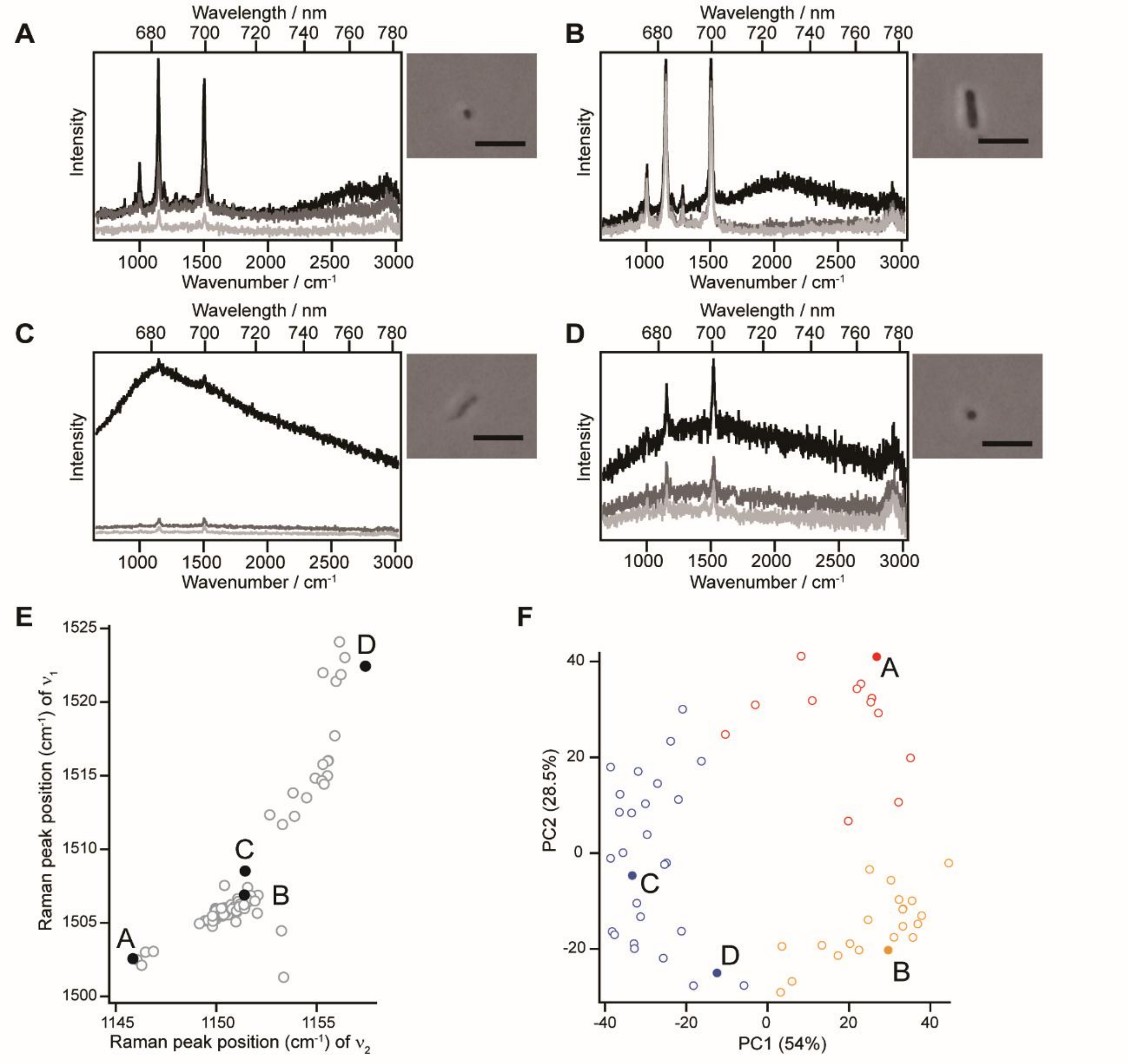


**Figure S4**

(**A-D**) Representative single-cell spectra of cells from clover leaves. The spectra were measured on quartz glass every 30 s up to 90 s (0–30 s, black; 30–60 s, gray; and 60–90 s, light gray lines). Scale bar = 5 μm. (**E, F**) Location of cells **A-D** in the distribution of the C=C stretching ($\nu_1$) peak and C–C stretching ($\nu_2$) peak positions of carotenoids (**E**) and in the principal component analysis score plot based on the autofluorescence spectra of cells from clover leaves (**F**).

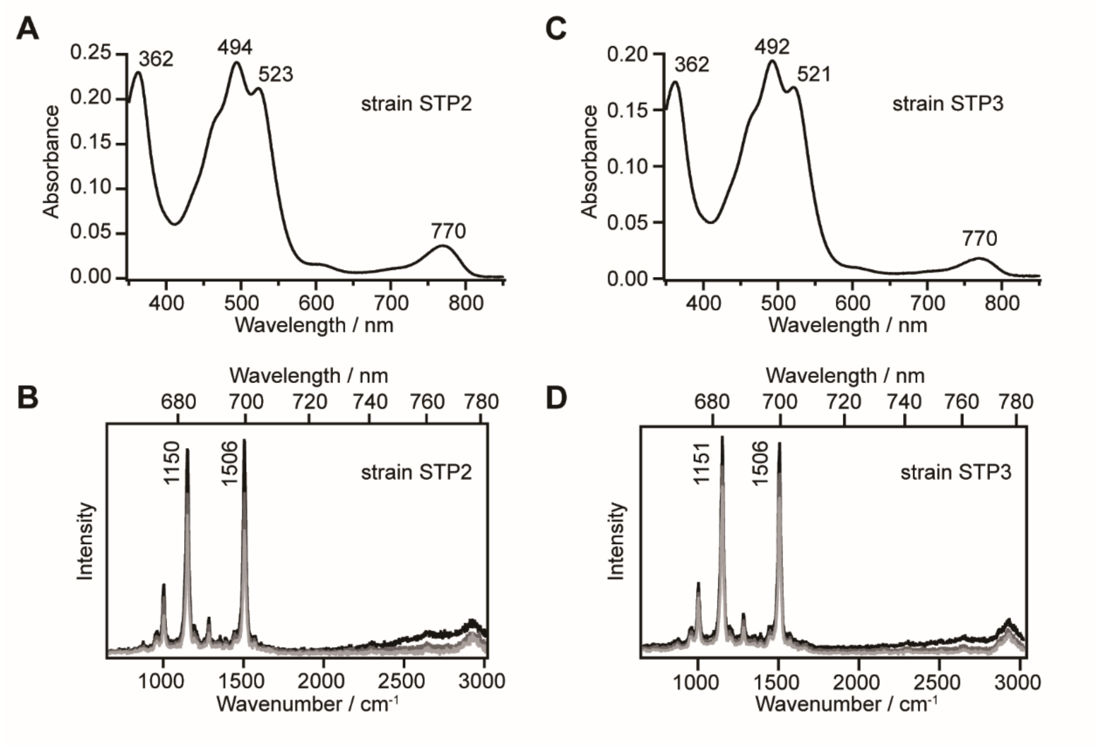


**Figure S5**

Absorption spectra of the pigment extracted from strain STP2 (**A**) and STP3 (**C**) from clover leaves and single-cell Raman and autofluorescence spectra of strains STP2 **(B**) and STP3 (**D**). The spectra in **B** and **D** were obtained in the same manner as in Fig. 1. For the electronic absorption spectra measurement (**A**, **C**), the cells of strains STP2 and STP3 cultured on an R2A agar plate were harvested with a sterile cell scraper and the pigments were extracted from the cells with methanol. The absorption spectra of the methanol extractions were recorded using a JASCO V-750 UV/Vis spectrophotometer (JASCO Co., Tokyo, Japan).
